# A young female presenting with heart failure secondary to eosinophilic myocarditis: a case report and review of the literature

**DOI:** 10.1186/s13104-018-3273-1

**Published:** 2018-03-09

**Authors:** Dissanayake Mudiyanselage Priyantha Udaya Kumara Ralapanawa, Kulatunga Wijekoon Mudiyanselage Pramitha Prabhashini Kumarihamy, Miriyalini Sundararajah, Widana Arachchilage Thilak Ananda Jayalath

**Affiliations:** 1 Department of Medicine, University of Peradeniiya, Peradeniya, Sri Lanka; 2 University Medical Unit, Teaching Hospital Peradeniya, Peradeniya, Sri Lanka

**Keywords:** Eosinophilic myocarditis, Heart failure, Idiopathic hypereosinophilic syndrome, Systemic steroids, Young female, Sri Lanka

## Abstract

**Background:**

Eosinophilic myocarditis is one of the fatal complications of idiopathic hypereosinophilic syndromes. Given the rarity of this form of myocarditis, it is often under-recognized. We describe a young girl who presented with features of heart failure. To our knowledge, this is the first reported case of eosinophilic myocarditis in a young Sri Lankan female.

**Case presentation:**

A previously healthy 21 year old Sri Lankan female admitted with shortness of breath for 1 week duration with associated low grade fever and profuse sweating. She was mildly febrile and dyspnoeic with absent ankle oedema. She was tachycardic and had elevated Jugular venous pressure with negative Kussmaul sign. Blood pressure was 100/70 mmHg. Clinically there was no cardiomegaly and heart sounds were slightly muffled with gallop rhythm. Bilateral basal fine end inspiratory crackles and mild hepatosplenomegaly were noted. The laboratory examinations showed leucocytosis with severe eosinophilia with no abnormal cells. Her ESR, Troponin I and Brain natriuretic peptide were elevated with normal CRP and electrocardiogram showed sinus tachycardia with wide spread ST depression. Heart failure was evident on chest X-ray and 2D-echocardiogram showed global left ventricular hypokinesia with 40% ejection fraction and a thin layer of pericardial effusion. Mild hepatosplenomegaly without lymphadenopathy was detected in the ultrasound scan. Bone marrow biopsy showed hypereosinophilia with no evidence of bone marrow infiltration. FIP1L1–PDGFRA fusion transcript and BCR–ABL transcript were not detected. Secondary causes for hypereosinophilia were excluded and the diagnosis of idiopathic hypereosinophilic syndrome and eosinophilic myocarditis was made. She had good response to steroids clinically and biochemically with complete recovery of left ventricular function. She is now on steroid to be continued at least 6 months to 1 year.

**Conclusion:**

Eosinophilic myocarditis is a rare but fatal disease if left untreated. Hence clinicians should have high index of suspicion to diagnose eosinophilic myocarditis in clinical context of heart failure due to myocarditis. The diagnoses of eosinophilic myocarditis may often be challenged especially in a poor recourse setting. However available investigation should be used to diagnose this condition without delay. Early treatment with systemic steroids may prevent fatal outcome and therapies for this disease have yet to be validated in large prospective studies.

## Background

Hypereosinophilia is a condition resulting from various eosinophilic diseases, including systemic vasculitis, helminth infection, drug hypersensitivity or idiopathic hypereosinophilic syndromes (HES). HES affect men more frequently than women. Eosinophilic myocarditis (EM) is one of the fatal complications of this condition if left untreated [1]. To date, there are only around 30 case reports of EM published in medical literature [2]. It is often under-recognized among clinicians due to the rarity of this form of myocarditis especially in Asia Pacific regions. Our case demonstrates the occurrence of heart failure due to myocarditis apparently secondary to eosinophilia in a young female patient. To our knowledge, this is the first reported case of eosinophilic myocarditis in a young Sri Lankan female. In addition, our case implicates the response to steroid therapy with complete recovery of ventricular function.

## Case presentation

A previously healthy 21 year old Sri Lankan female university student admitted with shortness of breath for 1 week duration. Shortness of breath was mainly on exertion, however at the time of admission it was present even at rest. She had low grade fever for the last 1 week associated with malaise and profuse sweating. Her weight and appetite have been steady throughout. She was not on any long term medication and did not take medication for minor ailments in the recent past to suggest a drug induced hypersensitivity reaction. She does not have a history of conjunctivitis, rhinitis, sinusitis or allergy to any drug or food. She took worm treatment 6 month prior. History was negative for malignancy, thromboembolic disorders and connective tissue diseases. She denied family history or other risk factors for cardiovascular disease. She is a non alcoholic, non smoker and no history of illicit drug use. She was mildly febrile and dyspnoeic at rest. There was no associated pallor or icterus. Generalized oedema or ankle oedema was absent. Physical examination was negative for malignancy, thromboembolic disorders and connective tissue diseases. She was tachycardic with regular, low volume pulse at rate of 120 beats per minute. Jugular venous pressure was elevated 5 cm above the angle of Louis with negative Kussmaul sign. Her blood pressure was 100/70 mmHg on admission. The cardiac apex was at its normal position and heart sounds were slightly muffled with gallop rhythm. There were bilateral basal fine end inspiratory crackles. Firm, non tender mild hepatomegaly with mild splenomegaly were present. Otherwise her clinical examination was normal. Her full blood count revealed absolute rise in eosinophil count of 21.6 × 10^3^ per microliter (63.5%) with 34 leukocytes per microliter with normal platelet count, haemoglobin and red cell indices. Blood picture showed high total white cell count with severe eosinophilia with no abnormal cells. Her erythrocyte sedimentation rate was 60 mm in 1st h in the presence of normal C-reactive protein. Sinus tachycardia with wide spread ST depression was evident on electrocardiogram. Chest X-ray was normal other than the evidence of heart failure. 2D-echocardiogram showed global left ventricular hypokinesia with 40% ejection fraction and thin layer of pericardial effusion. There was no associated intra cardiac thrombus. Troponin I was elevated up to 35.4 ng/dL (< 1.0 ng/dL) and Brain natriuretic peptide was 1280.5 pg/mL. Her renal function, thyroid function, serum electrolyte, calcium, magnesium levels, lipid profile were all normal with normal liver function but elevated liver enzymes (both were at 3 times upper limit of normal). Both serum IgM and IgG were negative for Filaria, Toxoplasma and toxocara infection and stool examination was negative for parasites. Serology for Epstein-Barr virus, Cytomegalo virus and Mycoplasma were negative. Retroviral infection and tuberculosis were excluded. Her blood culture and autoimmune screen were negative. Mild hepatosplenomegaly without lymphadenopathy was detected in otherwise normal ultrasound scan. Bone marrow biopsy showed hypereosinophilia with no evidence of bone marrow infiltration by lymphoproliferative malignancy. FIP1L1–PDGFRA fusion transcript and BCR–ABL transcript were not detected. The patient declined to undergo a confirmatory endomyocardial biopsy. Coronary angiogram and other non invasive cardiac imaging were not performed due to the practical problems of getting them done as they were not freely available. Clinically detected heart failure was confirmed by 2D ECHO and biochemical markers. The presence of global left ventricular dysfunction was better explained with myocarditis than myocardial infarction as there should be multi territory ischaemia to explain it which was less likely in our patient given that she was young and did not had any cardiovascular risk factors. This would have been excluded in certainty with coronary angiogram if it was freely available to us. Peripheral blood eosinophilia pointed towards a probable cause of cardiac damage in an otherwise healthy young female without cardiovascular risk factors. Drug hypersensitivity as a cause of eosinophilia was excluded from history. Bone marrow examination confirmed the diagnosis of hypereosinophilia. Myeloproliferative hypereosinophilic syndrome can give rise to chronic eosinophilic leukaemia. The possibility of this was excluded by negative FIP1L1–PDGFRA and BCR–ABL gene transcription. Secondary causes for hypereosinophilia were excluded and the diagnosis of idiopathic hypereosinophilic syndrome and eosinophilic myocarditis was made depending on the available investigations. Normal thyroid function test, negative ANA, DsDNA, negative antibody tires of common viral infection were used to exclude other differential diagnosis. This young female was given supportive care and treatment for heart failure and monitored in the high dependency unit to observe for possible deterioration. Her blood pressure dropped to 80/60 mmHg on day four of the admission requiring inotropic support. She was treated with intravenous Noradrenaline 0.3 μg/kg/min initially through L/Internal jugular venous catheter and then it was titrated up to 0.5 μg/kg/min to maintain mean arterial pressure of 70 mmHg. As soon as the diagnosis of hypereosinophilic syndrome with eosinophilic myocarditis was made, she was started on methylprednisolone 1 g/day for 3 days and continued with prednisolone 1 mg/kg to a total dose of 50 mg. Her symptoms started responding after the 3rd dose of methylprednisolone. Clinical improvement was observed in terms of symptoms and other parameters like blood pressure and pulse rate. Noradrenaline was then tailed off gradually and stopped after 5 days of starting steroids. Eosinophil count started to drop on day 5 of steroid treatment and it was then 16.31 × 10^3^ per microliter. Troponin level gradually normalized over the period of the next 2 weeks. She was discharged after 3 weeks of hospital stay and by this time Eosinophil count was 1.11 × 10^3^ per microliter (7.5%) and 2D echocardiography showed ejection fraction of 50% with thin layer of pericardial effusion. Pericardial effusion completely resolved and left ventricular function became normal (EF 60%) in the follow up 2D-ECHO and eosinophil count was at just upper limit of normal (0.52 × 10^3^ per microliter) after 2 week of discharge from the hospital.

Steroid dose was started to taper off after 1 month when she was totally asymptomatic and having persistently normal eosinophil count and left ventricular function. She is now on long term maintenance dose of steroid of 5 mg daily and Steroid needs to be continued at least 6 months to 1 year. She is on regular clinic follow up with monitoring of blood counts.

## Discussion and conclusion

Myocarditis is a rare disease which has a high mortality rate if left untreated or if the treatment is delayed [3]. Moreover eosinophilic myocarditis (EM) is a variety of myocarditis, which is rare, characterized by focal or diffuse myocardial eosinophilic infiltration [2, 4]. Although the cause of EM is not always apparent, condition resulting from several peripheral eosinophilic diseases are identified; parasitic infestation, hypersensitivity to a drug or substance, systemic vasculitis, malignancies, transplant rejection and idiopathic hypereosinophilic syndrome [4–6].

In our case, causes for hypereosinophilia were looked for, including Myeloproliferative hypereosinophilic syndrome, and the conclusion of idiopathic hypereosinophilic syndrome (HES) was made depending on the negative possible secondary causes. HES is defined by absolute eosinophil count greater than 1.5 × 105/L lasting for more than 6 months (or death before 6 months associated with signs and symptoms of hypereosinophilic disease) in the absence of any known condition of hypereosinophilia and with evidence of multi-organ involvement directly attributable to the eosinophilia or otherwise unexplained in the given clinical context [4, 5, 7]. Above mentioned criteria have been modified recently, especially regarding the persistence of blood eosinophilia for 6 months and presence of definitive tissue damage. For patients with marked eosinophilia and obvious tissue damage, as in cardiac involvement, immediate therapy should be initiated without observing for 6 months to arrive at a definitive diagnosis [4]. According to the available medical literature with case series, cardiac involvement occurs in up to 40–50% of patients with HES [8]. Our patient had only the cardiac involvement and is fortunate enough not to succumb to any other organ involvement at presentation such as lung, gastrointestinal system, skin and nervous system.

Patients with EM may admit with various signs and symptoms including fever, weight loss, malaise, chills and flu-like illness. Acute coronary syndrome-like features, heart failure, arrhythmias (tachy or brady) and intra cardiac thrombi are the life threatening complications [4–6, 8]. However they may present with sudden cardiac death as well. The patient in this scenario presented with acute heart failure with associated nonspecific symptoms.

Even though there are no globally accepted guidelines for the diagnosis of EM, The Japanese Circulation Society Task Force Committee on Acute and Chronic Myocarditis published helpful guidelines for the diagnosis and treatment of EM [9]. The essential diagnostic features include eosinophilia > 500/μL, cardiac symptoms, elevated cardiac enzymes, electrocardiogram (ECG) changes, and cardiac dysfunction on echocardiography, especially in the presence of unremarkable coronary angiography.

However, the definitive diagnosis requires an endomyocardial biopsy despite limited sensitivity (50%) and specificity of the biopsy due to patchy involvement of the myocardium and significant inter-observer variability in the interpretation of biopsy specimens respectively [10–13]. Echocardiography, nuclear imaging with gallium67- or indium111-labeled antimyosin antibodies and MRI are the non-invasive cardiac imaging useful in the diagnosis of myocarditis [10, 14]. However, none of these imagines show specific features that help to establish the diagnosis of eosinophilic myocarditis with certainty unlike with endomyocardial biopsy [14]. Arima and colleagues reported the place of serum levels of eosinophil cationic protein (ECP), one of several toxic proteins derived from the eosinophilic degranulation, in EM. Even though peripheral blood eosinophilia is one of the diagnostic criteria of EM, it is not always correlated with the extent of tissue damages [15]. In this regards, ECP levels may be used as an objective parameter of organ damage other than a marker used in diagnosis and assessing treatment response [15]. The endomyocardial biopsy was refused by our patient and none of the other non invasive imaging modalities were available to us except echocardiography. There was no facility for ECP as well and the treatment was started on clinical grounds and on available investigations.

The initial treatment goal after the diagnosis of EM is to provide hemodynamic stability with standard heart failure medication with full cardio-pulmonary support depending on the severity of heart failure and early treatment with corticosteroids. Systemic corticosteroid is the mainstay of treatment for a patient with EM and it should be commenced as soon as possible [16–18]. The goal of steroid treatment in EM is to reduce eosinophil induced organ damage. However it is important to identify reversible and readily treatable aetiologies, such as parasitic infection or drugs causing hypersensitivity and they should be addressed early. Before initiation of immunosuppressant, active infection needs to be rule out, using viral PCR for instance, to avoid worsening burden of disease [10].

Treatment with corticosteroid in HES has been documented in a published multicentre retrospective analysis [17]. In accordance with this, 85% of patients after 1 month of monotherapy had complete or partial responses. And most patients remained on maintenance doses with a median of 10 mg prednisolone daily dose for 2 months to 20 years [17, 18]. Kawano and colleagues were the first to suggest initiation and maintenance doses of prednisolone based on disease severity in EM according to a recent retrospective case series done by them [2]. They proposed initial 1 g methylprednisolone pulse dose for patients with severe disease, who are unstable, as compared to 1 mg/kg/day of prednisolone for more stable patients. Then a small maintenance dose of prednisolone was given to prevent relapse. Even though some literature advocates with gradual tapering of corticosteroids treatment for ≥ 1 year, [19] the duration of treatment remains unknown. Moreover, there is a relative lack of evidence-based guidelines in the use, dose, duration of corticosteroids or need for maintenance therapy in patients with EM. The proper answers for this needs to be validated in large multicenter, randomized studies [16]. However the rarity of this disease may be a limiting factor. The patient presented with EM with cardiogenic shock who is refractory to corticosteroid therapy, the use of adjunct azathioprine (2 mg/kg) was suggested [20]. In agreement with the available medical literature, our patient demonstrated drastic and complete recovery with systemic steroids followed by gradual tapering without requiring other immunosuppressants. Steroid use was combined with ACE inhibitors and beta blockers.

Failure of early diagnosis of this rare and likely under-diagnosed subtype of myocarditis and the delay of therapy may result in irreversible myocardial damage leading to fatal outcomes. Therefore clinicians should have a high index of suspicion to diagnose this when a patient is presented with a given clinical context in the presence of peripheral eosinophilia. As endomyocardial biopsy is the gold standard test but not always possible, the diagnoses of EM may often be challenged. Moreover therapies for this disease have yet to be validated in large prospective studies. In our case, the results of relevant laboratory analyses especially the presence of peripheral eosinophilia in a young female, presented with heart failure without any cardiovascular risk factors, and complete recovery of clinical features and left ventricular dysfunction with normalization of peripheral eosinophil count following early treatment of corticosteroids led to the diagnosis of EM even under a poor resource condition.

## Abbreviations

EM: eosinophilic myocarditis
HES: hypereosinophilic syndrome
ECG: electrocardiogram
2D-Echo: 2 dimensional echocardiogram
ANA: anti nuclear antibody
Ds DNA: double stranded deoxyribonucleic acid
PCR: polymerase chain reaction
